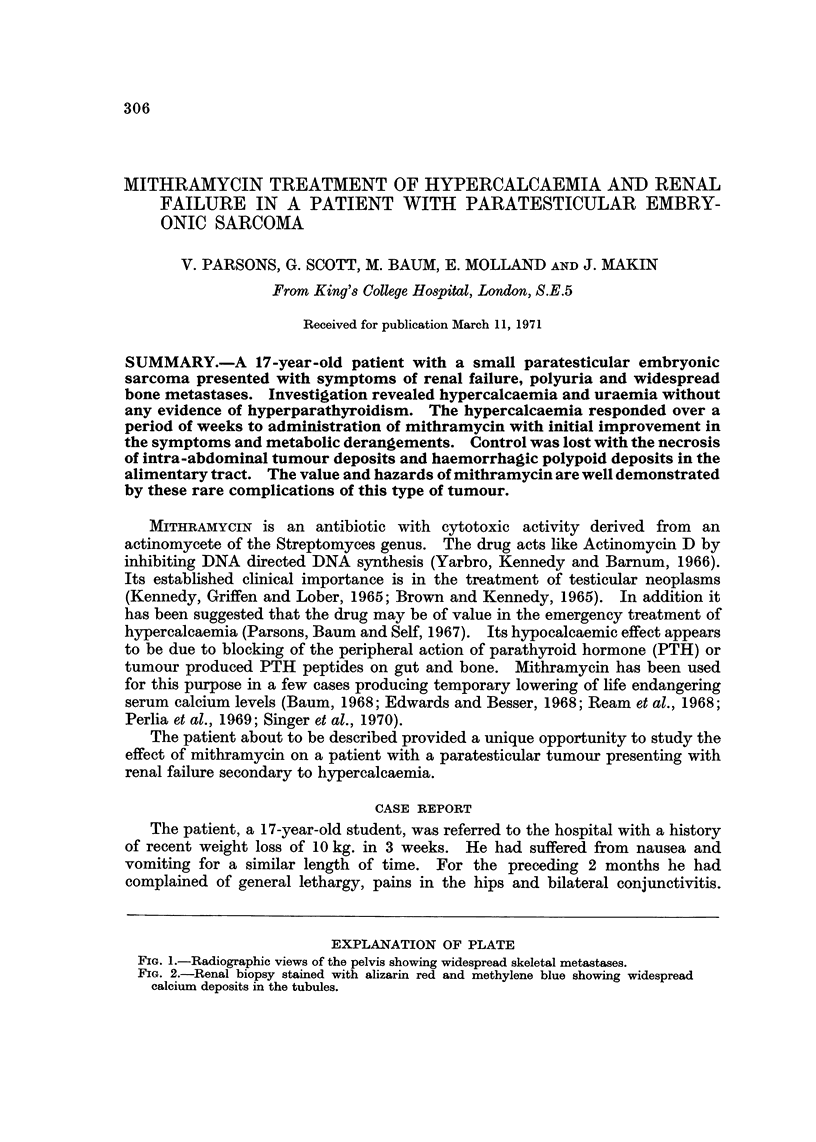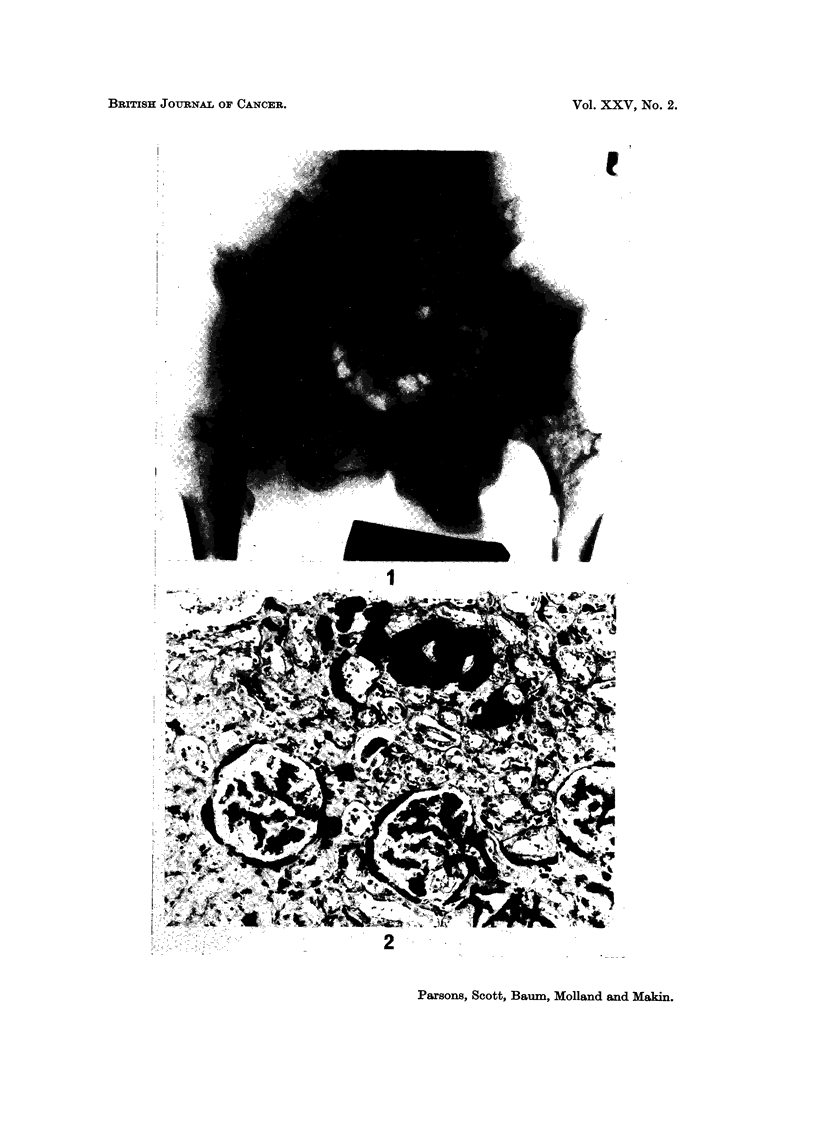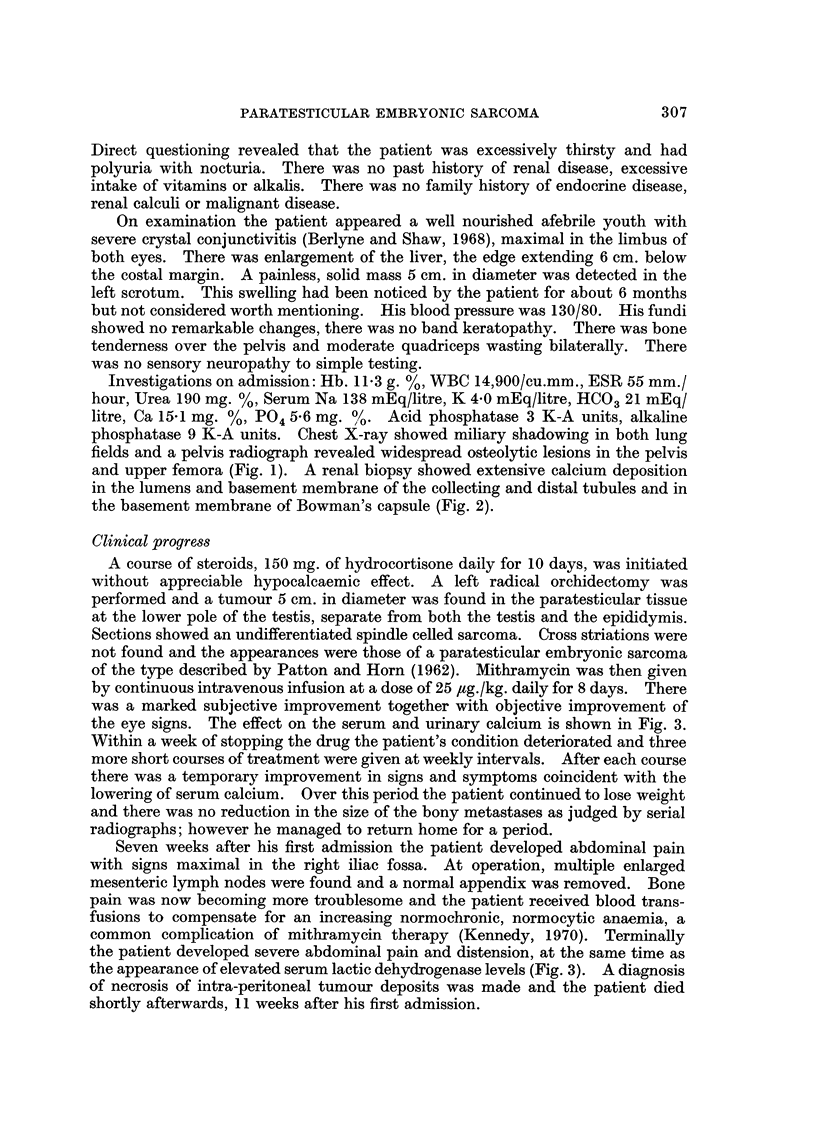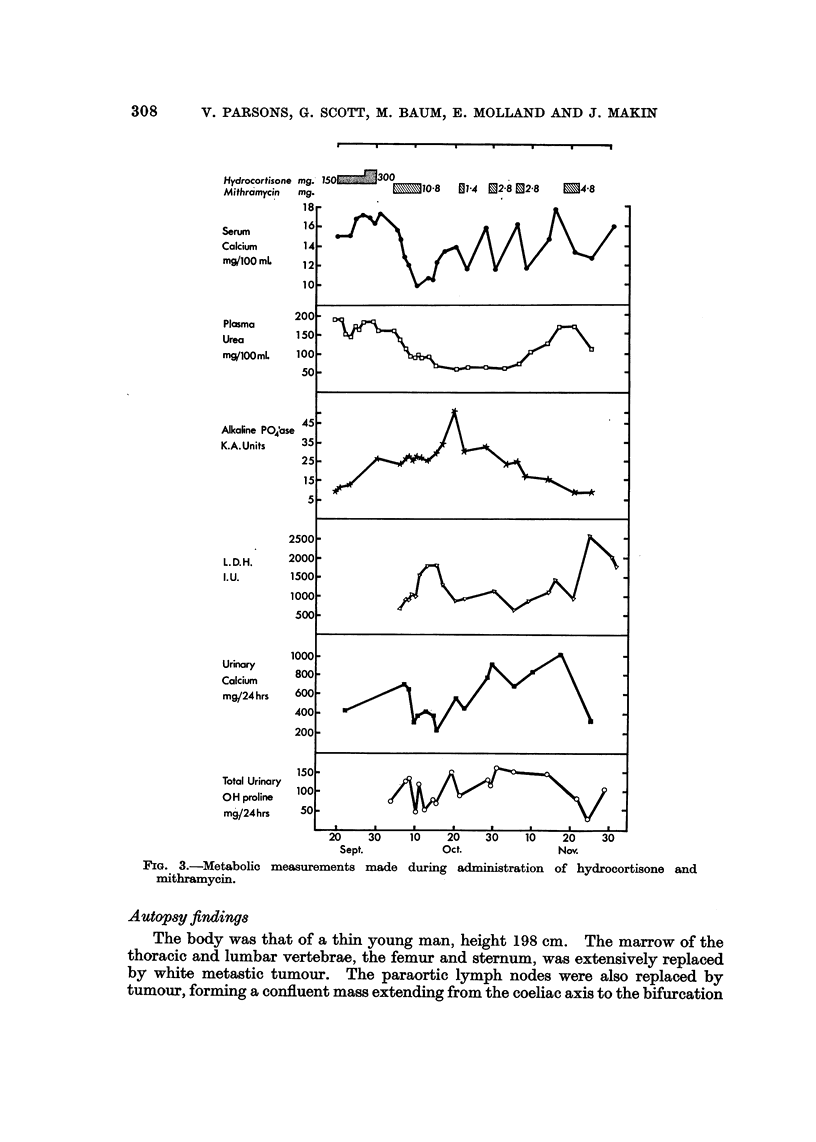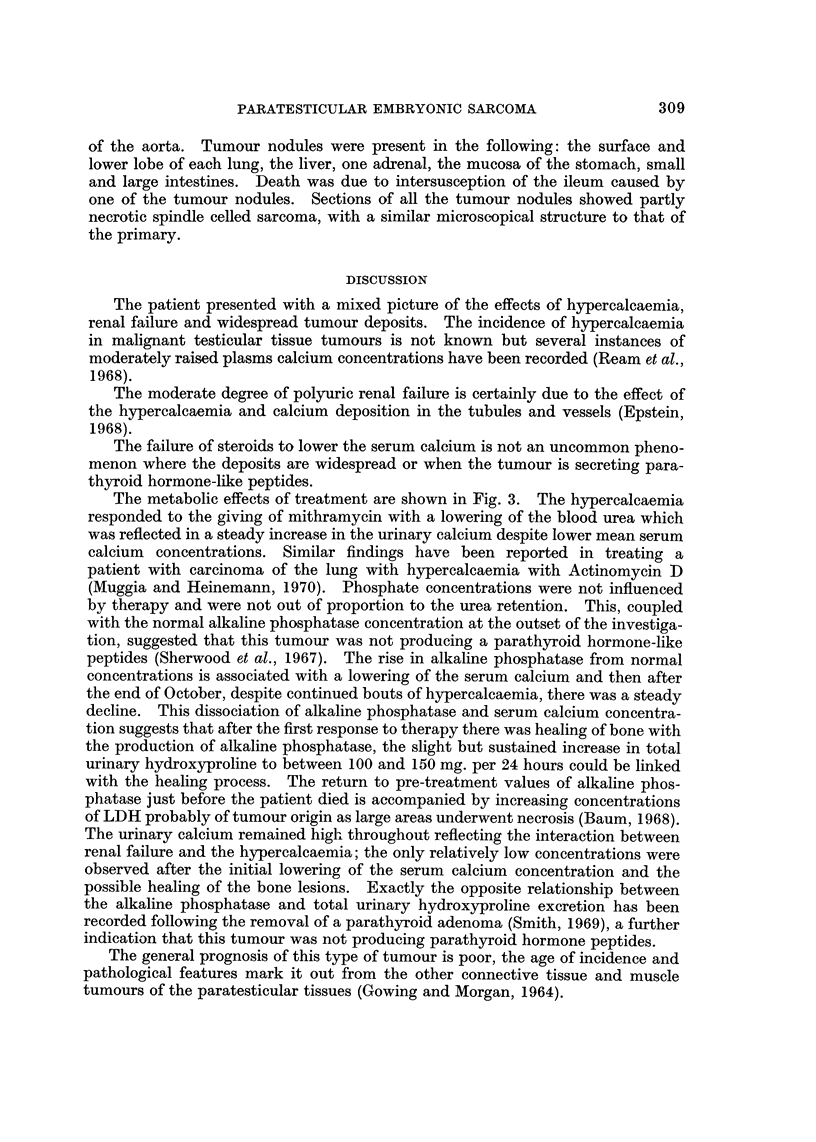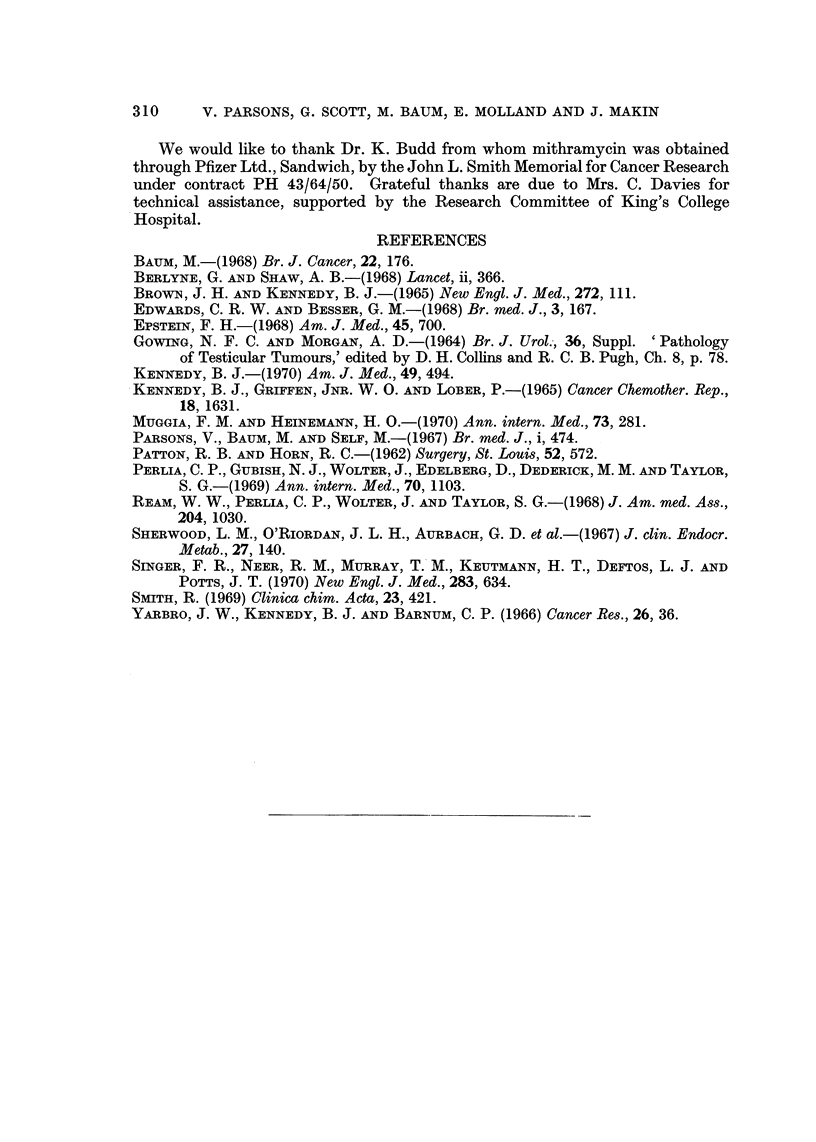# Mithramycin Treatment of Hypercalcaemia and Renal Failure in a Patient with Paratesticular Embryonic Sarcoma

**DOI:** 10.1038/bjc.1971.39

**Published:** 1971-06

**Authors:** V. Parsons, G. Scott, M. Baum, E. Molland, J. Makin

## Abstract

**Images:**


### MITHRAMYCIN TREATMENT OF HYPERCALCAEMIA AND RENAL FAILURE IN A PATIENT WITH PARATESTICULAR EMBRYONIC SARCOMA

V. PARSONS, G. SCOTT, M. BAUM, E. MOLLAND AND J. MAKIN

*From King's College Hospital, London, S.E.5*

Received for publication March 11, 1971

**SUMMARY.—A 17-year-old patient with a small paratesticular embryonic sarcoma presented with symptoms of renal failure, polyuria and widespread bone metastases. Investigation revealed hypercalcaemia and uraemia without any evidence of hyperparathyroidism. The hypercalcaemia responded over a period of weeks to administration of mithramycin with initial improvement in the symptoms and metabolic derangements. Control was lost with the necrosis of intra-abdominal tumour deposits and haemorrhagic polypoid deposits in the alimentary tract. The value and hazards of mithramycin are well demonstrated by these rare complications of this type of tumour.**

MITHRAMYCIN is an antibiotic with cytotoxic activity derived from an actinomycete of the Streptomyces genus. The drug acts like Actinomycin D by inhibiting DNA directed DNA synthesis (Yarbro, Kennedy and Barnum, 1966). Its established clinical importance is in the treatment of testicular neoplasms (Kennedy, Griffen and Lober, 1965; Brown and Kennedy, 1965). In addition it has been suggested that the drug may be of value in the emergency treatment of hypercalcaemia (Parsons, Baum and Self, 1967). Its hypocalcaemic effect appears to be due to blocking of the peripheral action of parathyroid hormone (PTH) or tumour produced PTH peptides on gut and bone. Mithramycin has been used for this purpose in a few cases producing temporary lowering of life endangering serum calcium levels (Baum, 1968; Edwards and Besser, 1968; Ream *et al.*, 1968; Perlia *et al.*, 1969; Singer *et al.*, 1970).

The patient about to be described provided a unique opportunity to study the effect of mithramycin on a patient with a paratesticular tumour presenting with renal failure secondary to hypercalcaemia.

### CASE REPORT

The patient, a 17-year-old student, was referred to the hospital with a history of recent weight loss of 10 kg. in 3 weeks. He had suffered from nausea and vomiting for a similar length of time. For the preceding 2 months he had complained of general lethargy, pains in the hips and bilateral conjunctivitis.

---

EXPLANATION OF PLATE

FIG. 1.—Radiographic views of the pelvis showing widespread skeletal metastases.

FIG. 2.—Renal biopsy stained with alizarin red and methylene blue showing widespread calcium deposits in the tubules.

1

2

Parsons, Scott, Baum, Molland and Makin.

Direct questioning revealed that the patient was excessively thirsty and had polyuria with nocturia. There was no past history of renal disease, excessive intake of vitamins or alkalis. There was no family history of endocrine disease, renal calculi or malignant disease.

On examination the patient appeared a well nourished afebrile youth with severe crystal conjunctivitis (Berlyne and Shaw, 1968), maximal in the limbus of both eyes. There was enlargement of the liver, the edge extending 6 cm. below the costal margin. A painless, solid mass 5 cm. in diameter was detected in the left scrotum. This swelling had been noticed by the patient for about 6 months but not considered worth mentioning. His blood pressure was 130/80. His fundi showed no remarkable changes, there was no band keratopathy. There was bone tenderness over the pelvis and moderate quadriceps wasting bilaterally. There was no sensory neuropathy to simple testing.

Investigations on admission: Hb. 11·3 g. %, WBC 14,900/cu.mm., ESR 55 mm./hour, Urea 190 mg. %, Serum Na 138 mEq/litre, K 4·0 mEq/litre, $HCO_3$ 21 mEq/litre, Ca 15·1 mg. %, $PO_4$ 5·6 mg. %. Acid phosphatase 3 K-A units, alkaline phosphatase 9 K-A units. Chest X-ray showed miliary shadowing in both lung fields and a pelvis radiograph revealed widespread osteolytic lesions in the pelvis and upper femora (Fig. 1). A renal biopsy showed extensive calcium deposition in the lumens and basement membrane of the collecting and distal tubules and in the basement membrane of Bowman's capsule (Fig. 2).

*Clinical progress*

A course of steroids, 150 mg. of hydrocortisone daily for 10 days, was initiated without appreciable hypocalcaemic effect. A left radical orchidectomy was performed and a tumour 5 cm. in diameter was found in the paratesticular tissue at the lower pole of the testis, separate from both the testis and the epididymis. Sections showed an undifferentiated spindle celled sarcoma. Cross striations were not found and the appearances were those of a paratesticular embryonic sarcoma of the type described by Patton and Horn (1962). Mithramycin was then given by continuous intravenous infusion at a dose of 25 $\mu$g./kg. daily for 8 days. There was a marked subjective improvement together with objective improvement of the eye signs. The effect on the serum and urinary calcium is shown in Fig. 3. Within a week of stopping the drug the patient's condition deteriorated and three more short courses of treatment were given at weekly intervals. After each course there was a temporary improvement in signs and symptoms coincident with the lowering of serum calcium. Over this period the patient continued to lose weight and there was no reduction in the size of the bony metastases as judged by serial radiographs; however he managed to return home for a period.

Seven weeks after his first admission the patient developed abdominal pain with signs maximal in the right iliac fossa. At operation, multiple enlarged mesenteric lymph nodes were found and a normal appendix was removed. Bone pain was now becoming more troublesome and the patient received blood transfusions to compensate for an increasing normochronic, normocytic anaemia, a common complication of mithramycin therapy (Kennedy, 1970). Terminally the patient developed severe abdominal pain and distension, at the same time as the appearance of elevated serum lactic dehydrogenase levels (Fig. 3). A diagnosis of necrosis of intra-peritoneal tumour deposits was made and the patient died shortly afterwards, 11 weeks after his first admission.

FIG. 3.—Metabolic measurements made during administration of hydrocortisone and mithramycin.

### *Autopsy findings*

The body was that of a thin young man, height 198 cm. The marrow of the thoracic and lumbar vertebrae, the femur and sternum, was extensively replaced by white metastic tumour. The paraortic lymph nodes were also replaced by tumour, forming a confluent mass extending from the coeliac axis to the bifurcation of the aorta. Tumour nodules were present in the following: the surface and lower lobe of each lung, the liver, one adrenal, the mucosa of the stomach, small and large intestines. Death was due to intersusception of the ileum caused by one of the tumour nodules. Sections of all the tumour nodules showed partly necrotic spindle celled sarcoma, with a similar microscopical structure to that of the primary.

### DISCUSSION

The patient presented with a mixed picture of the effects of hypercalcaemia, renal failure and widespread tumour deposits. The incidence of hypercalcaemia in malignant testicular tissue tumours is not known but several instances of moderately raised plasms calcium concentrations have been recorded (Ream *et al.*, 1968).

The moderate degree of polyuric renal failure is certainly due to the effect of the hypercalcaemia and calcium deposition in the tubules and vessels (Epstein, 1968).

The failure of steroids to lower the serum calcium is not an uncommon phenomenon where the deposits are widespread or when the tumour is secreting parathyroid hormone-like peptides.

The metabolic effects of treatment are shown in Fig. 3. The hypercalcaemia responded to the giving of mithramycin with a lowering of the blood urea which was reflected in a steady increase in the urinary calcium despite lower mean serum calcium concentrations. Similar findings have been reported in treating a patient with carcinoma of the lung with hypercalcaemia with Actinomycin D (Muggia and Heinemann, 1970). Phosphate concentrations were not influenced by therapy and were not out of proportion to the urea retention. This, coupled with the normal alkaline phosphatase concentration at the outset of the investigation, suggested that this tumour was not producing a parathyroid hormone-like peptides (Sherwood *et al.*, 1967). The rise in alkaline phosphatase from normal concentrations is associated with a lowering of the serum calcium and then after the end of October, despite continued bouts of hypercalcaemia, there was a steady decline. This dissociation of alkaline phosphatase and serum calcium concentration suggests that after the first response to therapy there was healing of bone with the production of alkaline phosphatase, the slight but sustained increase in total urinary hydroxyproline to between 100 and 150 mg. per 24 hours could be linked with the healing process. The return to pre-treatment values of alkaline phosphatase just before the patient died is accompanied by increasing concentrations of LDH probably of tumour origin as large areas underwent necrosis (Baum, 1968). The urinary calcium remained high throughout reflecting the interaction between renal failure and the hypercalcaemia; the only relatively low concentrations were observed after the initial lowering of the serum calcium concentration and the possible healing of the bone lesions. Exactly the opposite relationship between the alkaline phosphatase and total urinary hydroxyproline excretion has been recorded following the removal of a parathyroid adenoma (Smith, 1969), a further indication that this tumour was not producing parathyroid hormone peptides.

The general prognosis of this type of tumour is poor, the age of incidence and pathological features mark it out from the other connective tissue and muscle tumours of the paratesticular tissues (Gowing and Morgan, 1964).

We would like to thank Dr. K. Budd from whom mithramycin was obtained through Pfizer Ltd., Sandwich, by the John L. Smith Memorial for Cancer Research under contract PH 43/64/50. Grateful thanks are due to Mrs. C. Davies for technical assistance, supported by the Research Committee of King's College Hospital.

### REFERENCES

BAUM, M.—(1968) *Br. J. Cancer*, **22**, 176.
BERLYNE, G. AND SHAW, A. B.—(1968) *Lancet*, ii, 366.
BROWN, J. H. AND KENNEDY, B. J.—(1965) *New Engl. J. Med.*, **272**, 111.
EDWARDS, C. R. W. AND BESSER, G. M.—(1968) *Br. med. J.*, **3**, 167.
EPSTEIN, F. H.—(1968) *Am. J. Med.*, **45**, 700.
GOWING, N. F. C. AND MORGAN, A. D.—(1964) *Br. J. Urol.*, **36**, Suppl. 'Pathology of Testicular Tumours,' edited by D. H. Collins and R. C. B. Pugh, Ch. 8, p. 78.
KENNEDY, B. J.—(1970) *Am. J. Med.*, **49**, 494.
KENNEDY, B. J., GRIFFEN, JNR. W. O. AND LOBER, P.—(1965) *Cancer Chemother. Rep.*, **18**, 1631.
MUGGIA, F. M. AND HEINEMANN, H. O.—(1970) *Ann. intern. Med.*, **73**, 281.
PARSONS, V., BAUM, M. AND SELF, M.—(1967) *Br. med. J.*, i, 474.
PATTON, R. B. AND HORN, R. C.—(1962) *Surgery, St. Louis*, **52**, 572.
PERLIA, C. P., GUBISH, N. J., WOLTER, J., EDELBERG, D., DEDERICK, M. M. AND TAYLOR, S. G.—(1969) *Ann. intern. Med.*, **70**, 1103.
REAM, W. W., PERLIA, C. P., WOLTER, J. AND TAYLOR, S. G.—(1968) *J. Am. med. Ass.*, **204**, 1030.
SHERWOOD, L. M., O'RIORDAN, J. L. H., AURBACH, G. D. *et al.*—(1967) *J. clin. Endocr. Metab.*, **27**, 140.
SINGER, F. R., NEER, R. M., MURRAY, T. M., KEUTMANN, H. T., DEFTOS, L. J. AND POTTS, J. T. (1970) *New Engl. J. Med.*, **283**, 634.
SMITH, R. (1969) *Clinica chim. Acta*, **23**, 421.
YARBRO, J. W., KENNEDY, B. J. AND BARNUM, C. P. (1966) *Cancer Res.*, **26**, 36.